# Nicorandil potentiates sodium butyrate induced preconditioning of neurons and enhances their survival upon subsequent treatment with H_2_O_2_

**DOI:** 10.1186/s40035-017-0097-1

**Published:** 2017-10-30

**Authors:** Parisa Tabeshmehr, Haider Kh Husnain, Mahin Salmannejad, Mahsa Sani, Seyed Mojtaba Hosseini, Mohammad Hossein Khorraminejad Shirazi

**Affiliations:** 1 Student Research Committee, Shiraz University of Medical Sciences, Shiraz, Iran; 2 Cell & Molecular Medicine Student Research Group, Medical Faculty, Shiraz University of Medical Sciences, Shiraz, Iran; 3 Department of Basic Sciences, SRU, Riyadh, Saudi Arabia; 4 Stem Cell Laboratory, Department of Anatomy, Shiraz University of Medical Sciences, Shiraz, Iran

**Keywords:** Apoptosis, Neural stem cells, Oxidative stress, Preconditioning

## Abstract

**Background:**

Extensive loss of donor neural stem cell (NSCs) due to ischemic stress and low rate of differentiation at the site of cell graft are two of the major issues that hamper optimal outcome in NSCs transplantation studies. Given that histone deacetylases (HDACs) modulate various cellular processes by deacetylating histones and non-histone proteins, we hypothesized that combined treatment with small molecules, sodium butyrate (NaB; a known HDAC inhibitor) and nicorandil, will enhance the rate neuronal differentiation of NSCs besides their preconditioning to resist oxidative stress.

**Methods:**

NSCs derived from 14-day old Sprague Dawley rat ganglion eminence were characterized for tri-lineage differentiation. Treatment with 1 mM NaB significantly changed their culture characteristics while continuous treatment for 10 days enhanced their neural differentiation. NaB treatment also preconditioned the cells for their resistance to oxidative stress.

**Results:**

The highest rate of neural differentiation and preconditioning effect was achieved when the NSCs were treated concomitantly with NaB and nicorandil. Cell proliferation assay showed that concomitant treatment with NaB and nicorandil retarded their rate of proliferation.

**Conclusion:**

These data conclude that preconditioning of NSCs with NaB and nicorandil effectively enhances their differentiation capacity besides preconditioning the cells to support their survival under ischemic conditions.

**Electronic supplementary material:**

The online version of this article (10.1186/s40035-017-0097-1) contains supplementary material, which is available to authorized users.

## Background

Neural stem cells (NSCs) isolated from the adult and fetal nervous tissue, have been extensively studied for tri-lineage differentiation potential including neurons, oligodendrocytes and astrocytes in vitro as well as post-engraftment in experimental animal models [1]. One of the major problems during the experimental animal studies is the extensive apoptosis of donor NSCs post-engraftment due to the ischemic stress. It might also be the consequence of nutrient deprivation and oxidative load caused by the free radicals [2]. Regardless of the underlying cause, the altered oxidative load remains a significant determinant of the cell fate and function. The biochemical cues and inflammatory response emanating from the ischemic tissue activate redox-sensitive signaling pathways in the cells thus lowering the oxidative load to favor cell proliferation [3]. These molecular changes activate PI3K/Akt signaling via oxidative inactivation of PTEN thus promoting cell proliferation. On the contrary, as the concentration of reactive oxygen species (ROS) increases in the cells, the intracellular environment becomes more conducive for differentiation instead of supporting cell proliferation. This molecular mechanism holds true for both neural progenitor cells and glial precursor cells [4].

Histone deacetylases (HDACs) are modulators of gene expression profile and thus influence the various intracellular processes encompassing from survival to differentiation by deacetylating both histone and non-histone proteins [5]. Hence, treatment of NSCs with small molecule HDAC inhibitors (HDACi) exerts neuroprotective effects and stimulates neurogenesis [6, 7]. A series of small molecules including valproic acid (VPA), sub-eroylanilidec hydroxamic acids (SAHA), benzamide (MS-275), M344 and the short-chain fatty acid sodium butyrate (NaB) have been studied to modulate neural differentiation of stem cells [5, 8]. HDACi treatment of NSCs under pro-proliferation culture conditions leads to long-term changes in the cell fate in vitro by different mechanisms including inhibition of DNA synthesis [9] and by G1-phase arrest of the cell cycle [5]. HDACi also promote transcriptional changes in NSCs by increasing Cdk inhibitor genes p21 and p27 transcription and elevated H3K9 acetylation at proximal promoter regions of p21 and p27 [5].

Apurinic pyrimidinic endonuclease-1/Redox effector factor-1 (APE-1/Ref-1) is crucial for cellular response to oxidative stress [10]. This is achieved by N-terminus lysine reaction with the nucleic acids and nucleophosmin besides base excision repair through C-terminus initiating enzymatic activity. The anti-apoptotic function of APE1 under oxidative stress has been confirmed via activation of nuclear factor-kappa B (NF-kB) signaling [11, 12]. Nicorandil is a stimulator of the APE1 pathway in the neurons subjected to oxidative stress [13, 14]. The present study was designed to determine the combined protective effect of NaB and nicorandil on NSCs under oxidative stress. We hypothesized that serial treatment of NSCs with NaB (HDACi) followed by nicorandil (as the APE1 stimulator) would promote neuronal differentiation of NSCs that would be preconditioned to resist oxidative stress.

## Methods

The present study conformed to the Guideline for the Care and Use of Laboratory Animals and all the experimental animal procedures were performed strictly in accordance with protocol approved by Ethical Committee of Shiraz University of Medical Sciences, Iran. All surgical manipulations were carried out under general anesthesia. The results shown in the manuscript are replicate of five experiments.”

### NSCs isolation and culture

NSCs were obtained from 14-day old Sprague Dawley rat ganglion eminence as described earlier (Additional file 1). One week after isolation, sphere-like colonies of neurospheres were observed that were trypsinized as single cells and passaged into new culture flasks at 50000 cells/ml concentration (Additional file 1).

### Characterization of NSCs

NSCs were characterized for tri-lineage differentiation into neurons, astrocytes and oligodendrocytes as described earlier (Additional file 1).

### Preconditioning with histone deacetylase inhibitor

For the differentiation NSCs using HDACi alone, the cells were treated with freshly prepared 1 mM NaB in distilled water (Cat# B5887, Sigma Aldrich, St. Louis, USA). NaB treatment was performed at 2 h after NSCs passage as single cell culture. Neural differentiation of NSCs was assessed by flow cytometry and immunocytochemistry on day-7 after NaB treatment.

For flow cytometry, the cells were fixed with 4% paraformaldehyde for 20 min at 4 °C. Subsequently, the cells were incubated with MAP-2 specific primary antibody (1:1000, cat # ab5392, Abcam, Cambridge, UK) at room temperature. After one hour, the cells were washed with phosphate buffer saline (PBS) and the primary antigen-antibody reaction was detected by incubating the cells for 1 h at room temperature with fluorescently conjugated secondary antibody diluted in 5% goat serum. The MAP-2 positive cells were analyzed by flow cytometry (BD Bioscience, San Jose, USA). For microscopic analysis of MAP-2 positive cells, the cells were stained with 4′,6-diamidino-2-phenylindole (DAPI; 1:1000; Millipore S7113, Billerica, USA) and observed using fluorescence microscope (Olympus BX53; Tokyo, Japan). The MAP-2 positive neurons were counted in various microscopic fields and compared with the untreated control group.

### Cell cluster and neurosphere count

The NSCs derived cell clusters and neurospheres were counted as described earlier [5]. One week after HDACi treatment, 10 randomly selected microscopic fields were counted using an inverted microscope (Olympus; Tokyo, Japan). The inclusion criteria was set as small cell cluster an aggregation of cell count > 4 cells but cell aggregation diameter of < 50 μm. For neurospheres, the diameter was set as more than 50 μm. The number of small cell clusters and neurospheres were compared with the control group [5].

### Cell proliferation assay

5-Bromo-2′-deoxyuridine (BrdU) immunocytochemistry was performed to assess NSCs proliferation after NaB treatment. In brief, after NaB treatment, 1 × 10^5^ cells in 96-well plates were incubated with 25 μM BrdU (Cat #B5002; Sigma-Aldrich; St.Louis, USA). After 16 h, the cells were washed and fixed with 4% paraformaldehyde for 20 min at 4 °C and later treated with 1 N HCl for 15 min at 37 °C. Subsequently, the cells were incubated with anti-BrdU specific primary antibody (Cat# B8434; 1:1000; Sigma-Aldrich; St. Louis, USA) in 0.1% triton and 2.5% BSA in PBS. The cells were kept at room temperature for 2 h followed by washing ×3 with PBS. The primary antigen-antibody reaction was detected with the Alexa flour-488 conjugated secondary antibody. The cells were incubated with the secondary antibody at 37 °C for 1 h, washed and observed using fluorescence microscope (Olympus BX53; Tokyo, Japan). Cell proliferation was measured by BrdU+/total number of cells in NaB treated group.

The data was analyzed by unpaired *t*-test using Prism 8.00 software.

### Treatment with nicorandil

To determine the activation of the APE1 pathway, 12.5 μM nicorandil was added to the cells as described earlier [14].

### Induction of apoptosis using H_2_O_2_

For induction of apoptosis, 500 μM H_2_O_2_ was added to the culture media for one hour at one hour after nicorandil treatment [15].

### MTT assay

The viability of the preconditioned and control cells were assessed by MTT assay at 24 h after their exposure to H_2_O_2_. Briefly, NSCs culture media in different treatment groups was supplemented with 10 μl of 5 mg/ml 3-(4,5-Dimethyl-2-thiazolyl)-2,5-diphenyl-2H-tetrazolium bromide (MTT) (Cat# M2128; Sigma-Aldrich; St. Louis, USA). The cells were then incubated at 37 °C for 4 h. At the end of the incubation period, the medium was removed, treated with acidic isopropanol (0.1 N HCl in isopropanol) and the samples were read using spectrophotometer at wavelength 570 nm [16]. The survival of the cells in control group (which was exposed to H_2_O_2_ without small molecule treatment) was considered as 100% and the other treatment groups were compared with the control. In accordance with the calculations, group survival of more than 100% represents the treatment as protective against H_2_O_2_ stress.

### Annexin-V staining and flow cytometry

The resistance of the HDACi induced neurons to H_2_O_2_ treatment was assessed by flow cytometry using Annexin-V Apoptosis detection kit in combination with propidium iodide (PI) according to manufacturer’s instructions (Cat# 14085; Abcam; Cambridge, UK). Briefly, 5 × 10^5^ cells with or without HDACi treatment were incubated with Annexin V-FITC/PI for 1 h at room temperature. After washing ×3 with PBS, the cells were harvested using Trypsin-EDTA. After washing ×2 with PBS, the cells were analyzed by flow cytometry (BD Bioscience, San Jose, USA) in FITC channel (488). The cells were stained with PI to discriminate the necrotic cells from the apoptotic cells and measured by flow cytometry. The apoptotic cells were defined as the ones staining positive for Annexin-V.

### Colorimetric analysis and immunocytochemistry for caspase-3

The activity of caspase-3 was measured using caspase-3 Assay kit (Cat# ab39401; Abcam, Cambridge, USA) according to the manufacturer’s instructions. Immunocytochemistry for caspase 3 was performed with anti-caspase-3 antibody (Cat# MAB10753; Sigma Aldrich; St. Louis, USA**)**.

### Statistical analysis

Data were presented as mean ± STD. For quantitative analysis, data was analyzed with unpaired t-test and one-way ANOVA with post-hoc analysis using SPSS 16.00. A value of *p*˂0.05 was considered as statistically significant.

## Results

### NSCs culture and characterization

**Fig. 1:**
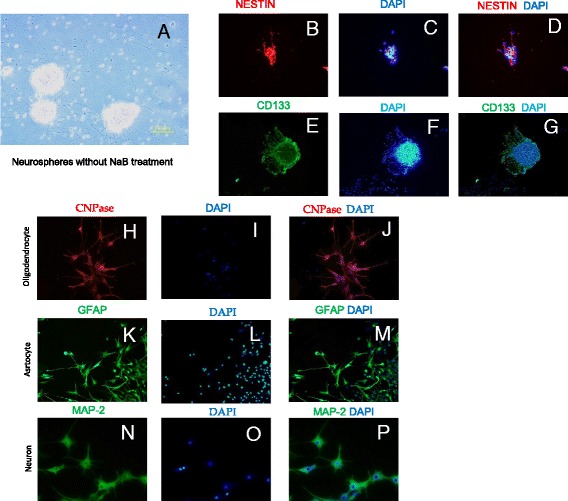
Neural stem cell culture and characterization. **a** Phase contrast image of neural stem cells (NSCs) derived aggregates of neurospheres after 5–7 days of in vitro culture in NuroCult NS-A medium. The cells were maintained at 37 °C and 5%CO_2_ culture conditions. Immunocytochemistry of NSCs for CD133 (**b**-**d**) and Nestin expression (**e**-**g**) using specific antibodies for the respective antigen. Differentiation of NSCs to oligodendrocyte and astrocyte was assessed by immunostaining for CNPase (Oligodendrocytes specific marker; **h**-**j**) and GFAP (astrocytes specific marker; **k**-**m**) expression using specific antibodies for the respective antigen. The cells were subjected to tri-lineage differentiation assay in vitro. (**n**-**p**) The neural differentiation of NSCs was confirmed by MAP-2 antibody staining

The isolated NSCs were cultured in NeuroCult NS-A media (Cat# 05750; Stem Cell Technology, Vancouver; Canada) and maintained at 37 °C and 5% CO_2_. After 5 days in the culture, NSCs started to form spherical cell aggregates or the neurospheres (Fig. 1a). The spherical cell aggregates were clearly distinguishable from the extraneous particulate material in the culture based on their morphology at higher magnification. Fluorescence immunocytochemistry with specific primary antibodies revealed their positivity for both Nestin (Fig. 1b-d) and CD133 expression (Fig. 1e-g). The neurospheres were dissociated into single cells and cultured in differentiating media for tri-lineage differentiation assay. Fluorescence immunocytochemistry showed their successful differentiation into neurons; oligodendrocytes and astrocytes as was evident from positivity for CNPase (for oligodendrocytes) and GFAP (for astrocytes) (Fig. 1h-j and k-m).

### NSCs sphere formation and proliferation

**Fig. 2:**
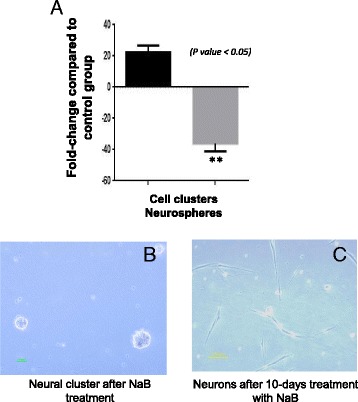
Sodium butyrate (NaB) treatment of neural stem cells (NSCs). **a** Treatment of NSCs with NaB decreased the rate of neurosphere formation whereas the rate of cluster formation was significantly increased as compared to the non-treated control cells (*P < 0.05*). **b** Phase contrast image of neural clusters after treatment with NaB. **c** Phase contrast image of the derivative neurons after 10-day treatment with NaB

**Fig. 3:**
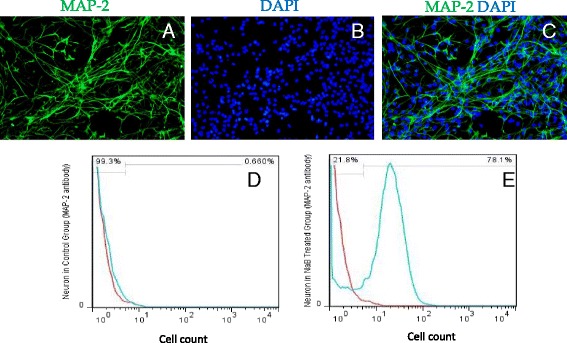
Sodium butyrate (NaB) enhances neural differentiation of neural stem cells (NSCs). **a**-**c** Immunocytochemistry of NSCs for neural differentiation using MAP (green fluorescence) as neuron specific marker using specific antibody. The nuclei were visualized using DAPI (blue fluorescence). **d**, **e** Flow cytometeric analysis of derivative neurons to ascertain the rate of neuronal differentiation which increased significantly subsequent to NaB. Up to 78.1% NSCs were differentiated after NaB treatment as compared to the non-treated (0.66%) controls

Treatment with NaB clearly changed the culture characteristics of NSCs in comparison with the untreated control NSCs that were maintained under similar culture conditions. The most obvious difference between the two groups of cells was that NaB treatment markedly reduced their ability to form neurospheres despite significantly enhanced rate of cluster formation (*p < 0.05* vs control) (Fig. 2a-b). A continuous treatment for 10 days promoted neuronal differentiation of the clusters which had elongated morphology with distinct cell body, dendrite and axon (Fig. 2c). Neural differentiation was confirmed by MAP-2 expression using fluorescence immunocytochemistry (Fig. 3a-c) and flow cytometry (Fig. 3d-e). As compared to the control non-treated cells, 78.1% cells differentiated into neurons after 10 days treatment with NaB (Fig. 3d-e). The flow cytometry data showed that NaB treatment for HDAC inhibition significantly enhanced the neural differentiation.

### Cell viability

**Fig. 4:**
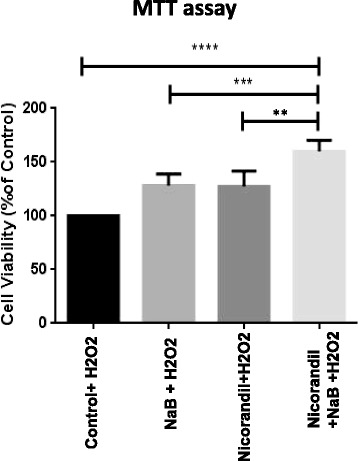
Sodium butyrate (NaB) treatment preconditions NSCs. MTT assay showed that that viability of NaB treated NSCs increased significantly upon exposure to oxidative stress as compared to the non-treated control cells. The group of cells with combined treatment of NaB and Nicorandil showed the highest rate of cell viability after H_2_O_2_ exposure as compared to the other groups (*p < 0.05* vs all other groups of cells)

An interesting feature of our study was that NaB treatment preconditioned the cells towards subsequent exposure to H_2_O_2_. The viability of NaB treated cells was significantly higher as compared to the non-preconditioned cells upon subsequent exposure to H_2_O_2_. However, preconditioning with NaB in the presence of nicorandil significantly enhanced the cell viability further as compared to the control as well as NaB alone treated cells as assessed by MTT assay (*p < 0.05*; Fig. 4).

### Caspase-3 activity and annexin-V assays

**Fig. 5:**
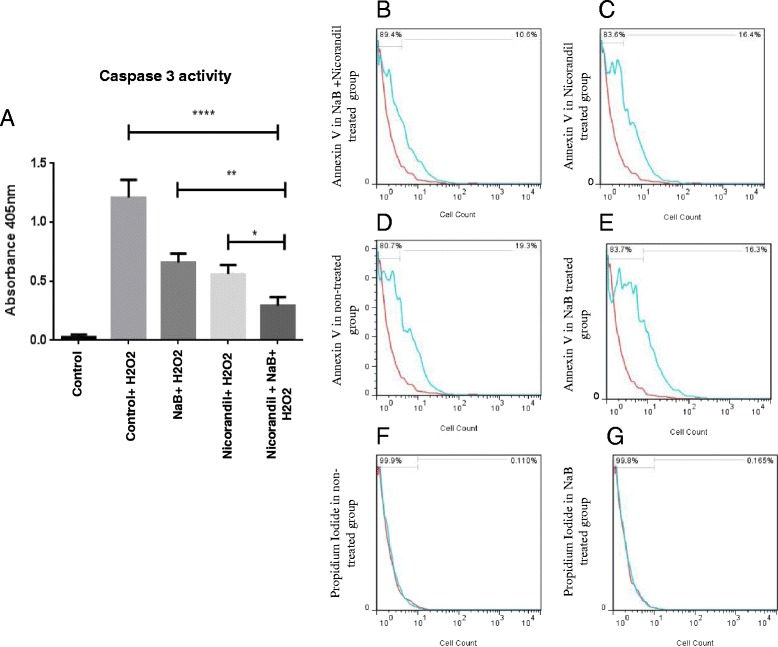
Preconditioning effect of combined treatment of NSCs with Sodium butyrate (NaB) and Nicorandil. Combined treatment with NaB and nicorandil significantly reduced NSCs apoptosis upon subsequent exposure to oxidative stress could diminish the apoptosis after stress oxidative exposure. **a** Caspase 3 activity was significantly higher in the untreated NSCs after exposure to oxidative stress whereas preconditioning with either NaB or nicorandil treatment alone significantly reduced caspase 3 activity. Lowest caspase 3 activity was observed in the cells which had combined pre-treatment with NaB and Nicorandil. **b**-**e** Similarly, Annexin V assay showed lowest apoptosis in the combined (NaB and nicorandil) treatment group. Untreated cells without exposure to oxidative stress were used as baseline control. Propidium iodide staining showed that the more than 99% cell death was because of the apoptosis and not due to necrosis (**f**-**g**)

Caspase-3 plays an important role in cell apoptosis and initiates the execution-phase of the apoptosis [17]. Hence, caspase-3 activity was measured to identify the apoptotic cells in different treatment cell groups upon exposure to H_2_O_2_ (Fig. 5a). The caspase-3 activity was significantly higher in the non-treated cell group upon exposure to H_2_O_2_ as compared to the NaB and combined (NaB + nicorandil) treatment groups whereas least activity of caspase-3 was observed in the combined (NaB + nicorandil) treatment group. Untreated cells without exposure to H_2_O_2_ were used as baseline control (Fig. 5a). These results were well supported by Annexin-V flow cytometry assay which showed that the percentage of Annexin-V positive cells was 19.3% in the untreated control cells upon exposure to oxidative stress as compared to 16.3% in NaB preconditioned cells and 10.6% in the combined (NaB and nicorandil) treatment group (*p < 0.005* vs control; Fig. 5b-g). PI staining combined with flow cytometry showed that the more than 99% cell death was due to apoptosis (Fig. 5f-g).

### Cell proliferation assay by BrdU labeling

**Fig. 6:**
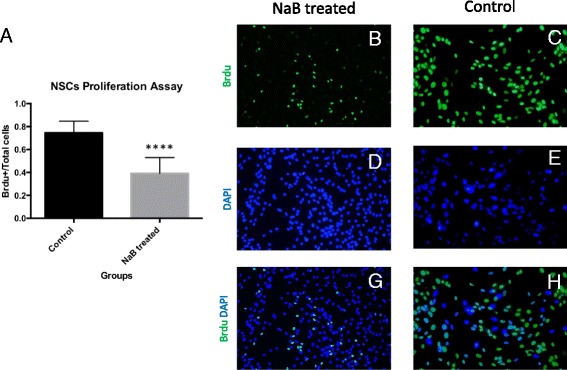
Cell proliferation assay. The cell proliferation assay using BrdU labeling showed that treatment of sodium butyrate (NaB) attenuated the rate of neural cell proliferation. There was significant difference between the NaB treated and untreated control groups (*p < 0.05*). (**a**) Quantitative assessments of Brdu positive cells. (**b**-**h**) Proliferating cells in different groups

To assess the proliferation of NSCs after NaB treatment, the cells were labeled with BrdU. The number of the NaB treated cells positive for (Brdu^+^/total cells) was (1.06 ± 0.04) as compared to the untreated control group (1.18 ± 0.10; *p < 0.05*) (Fig. 6).

## Discussion

The main finding of our study is that NSCs treated with NaB successfully produce preconditioned neurons while subsequent treatment with nicorandil accentuates the preconditioning effect of NaB and enhances their survival upon subsequent exposure to H_2_O_2_.

The rate of neuronal differentiation of NSCs without teratogenicity and the ability of the differentiated cells to survive in the ischemic environment post-engraftment are two of the major challenges in stem cell-based therapy [18, 19]. Spontaneous malignant transformation of NSCs after long-term culture has been attributed to their extensive proliferation potential [20]. Molecular studies have shown constitutively higher NFkB activity, enhanced VEGF expression and adoption of tumorgenic phenotype in NSCs under growth factor-free culture conditions (in the absence of EGF and bFGF) [21]. Neutralization of VEGF significantly reduces the proliferation potential of NSCs in the growth factor-free culture and promotes their differentiation [22]. Various strategies have been adopted to promote their rate of neuronal differentiation. For example, alleviation of hypoxia in the developing cortex through angiogenesis promotes neurogenic differentiation of NSCs [23]. Similarly, exploiting the critical role of cell cycle regulators to prepare the cells for differentiation via cell cycle exit, double knock-down of cyclin dependent kinase (cdk)2 and cdk4 significantly enhances neuronal differentiation of neuronal precursors both in vivo and in culture conditions [24]. Supported by the loss-of-function studies, both cell proliferation and survival are significantly affected by the class-I HDACs [25, 26]. HDACi treatment stops the proliferation and sphere-forming potential of NSCs and supports their neural differentiation [27–29]. These data vividly support our findings that treatment with NaB enhances neural differentiation of NSCs. We observed that NaB effectively suppressed the rate of NSCs proliferation as was evident from increased cell cluster formation and their reduced sphere-forming ability. At molecular levels, previous studies have shown that NaB inhibits deacetylation of the lysine and arginine residues on the N-terminus of histones with concomitant increase in p21 which is responsible for anti-proliferative effects of the HDACi [30–33]. Their exit from cell cycle is accompanied by change of fate as a result of which NSCs become committed to tri-neural lineage fate, with neural differentiation as the dominant prospect [8, 34]. A recent study has shown that pro-neural genes *Ngn2* and *NeuroD1* are elevated during HDACi treatment with a consequent increase in neural differentiation [35]. Our results are in agreement with the published reports and show that NaB induce significantly higher neural differentiation of NSCs in comparison with the non-treated control NSCs as determined by MAP-2 antigen expression.

Besides exit from cell cycle and neural differentiation, cytoprotection afforded by NaB was the cardinal feature of our study. The NaB treated cells were more resistant to H_2_O_2_ induced apoptosis than the untreated control cells (*p < 0.05* vs untreated control cells) as determined by MTT and caspase-3 assays. Neuroprotective effects of NaB have also been reported in the retinal glial cells cultured under serum-free conditions at par with erythropoietin [36]. Mechanistic studies have shown that cytoprotection with NaB was mediated via activation of PI3K/Akt signaling [37]. Treatment with NaB significantly reduced the number of apoptotic neurons during cerebral ischemia-reperfusion injury in experimental mouse model. The authors have reported significantly elevated Bcl2 and phosphorylated Akt, and reduced caspase-3 and Bax expression as compared to the non-treated experimental animals. Enhanced resistance to oxidative stress has also been reported in rat hippocampus via elevated levels of thiorexidine binding protein-2 (TBP-2) after NaB treatment which was attributed to antioxidant effects of TBP-2 [38]. We observed that combined treatment of NSCs with NaB and nicorandil was more cytoprotective as compared to treatment of the cells with either of the two molecules alone. Nicorandil is a small molecule capable of stimulating APE1/Ref1 activity in the cells which is responsible for cell reaction to DNA damage and oxidative stress [39]. The cytoprotective effects of APE1/Ref1 have been attributed to modulation of transcription factors including activator protein-1 (AP-1), NF-ĸB, p53, cAMP response element binding protein (CREB), and hypoxia-inducible factor-1α (HIF-1α) [14, 40, 41]. Based on these data, we propose that nicorandil treatment activated APE1 in the neurons after treatment with HDACi. Further studies are warranted to fully understand the underlying molecular mechanism and efficacy of the combined cytoprotective effects of NaB and nicorandil.

Notwithstanding the interesting data, the study has some limitations. Firstly, we focused only on the apoptosis markers for evaluation of the preconditioning effects of NaB and nicorandil. Although H_2_O_2_ has been widely accepted as an inducer of apoptosis in various cell-based studies, it may induce cell death by multiple mechanisms [42–44]. Study of the oxidative stress markers would have enhanced the significance of the data. Secondly, future studies will be required to understand the mechanism of cytoprotection afforded by the combined treatment with NaB and nicorandil using HDAC-mutant cells, loss-of-function and gain-of-function studies to understand the role of p21 in proliferation of the preconditioned cells besides their cell cycle marker expression profile.

## Conclusion

In conclusion, combined treatment with NaB and nicorandil is effective to enhance neural commitment of NSCs and cytoprotection of the derivative neural cells.

## Additional file

Additional file 1: (a-b) A conceptual look at how NaB and Nicroandil preconditioning increases neural cell viability upon exposure to oxidative stress. (DOCX 18 kb)

## Abbreviations

APE-1: Apurinic pyrimidinic endonuclease-1
bFGF: Basic fibroblast growth factor
cGMP: cyclic guanosine-3′,-5′-monophasphate
EGF: Epidermal growth factor
HDACi: Histone deacetylases inhibitors
HDACs: Histone deacetylases
iPSCs: Induced pluripotent stem cells
MCAO: Middle cerebral artery occlusion
MS-275: Benzamide
NaB: Sodium butyrate
NSCs: NSCs
PBS: Phosphate buffered saline
Ref-1: Redox effector factor-1
SAHA: Assuberoylanilidec hydroxamic acid
TUNEL: dUTP nick end labeling
VEGF: Vascular endothelial growth factor
VPA: Valproic acid

## References

[1] Zhao Y, Zuo Y, Jiang J, Yan H, Wang X, Huo H (2016). Neural stem cell transplantation combined with erythropoietin for the treatment of spinal cord injury in rats. Exp Thera Med.

[2] Lee IH, Huang SS, Chuang CY, Liao KH, Chang LH, Chuang CC, Su YS, Lin HJ, Hsieh JY, Su SH, Lee OK (2017). Delayed epidural transplantation of human induced pluripotent stem cell-derived neural progenitors enhances functional recovery after stroke. Sci Rep.

[3] Bjugstad KB, Rael LT, Levy S, Carrick M, Mains CW, Slone DS (2016). Oxidation-reduction potential as a biomarker for severity and acute outcome in traumatic brain injury. Oxid Med Cell Longev.

[4] Lu J, Xie L, Liu C, Zhang Q, Sun S. PTEN/PI3k/AKT regulates macrophage polarization in emphysematous mice. Scand J Immunol. 2017;85(6):395-405. doi:10.1111/sji.12545. 10.1111/sji.12545 28273403

[5] Zhou Q, Dalgard CL, Wynder C, Doughty ML (2011). Histone deacetylase inhibitors SAHA and sodium butyrate block G1-to-S cell cycle progression in neurosphere formation by adult sub-ventricular cells. BMC Neurosci.

[6] Hsing CH, Hung SK, Chen YC, Wei TS, Sun DP, Wang JJ (2015). Histone deacetylase inhibitor trichostatin a ameliorated endotoxin-induced neuro-inflammation and cognitive dysfunction. Mediat Inflamm.

[7] Ziemka-Nalecz M, Jaworska J, Sypecka J, Polowy R, Filipkowski RK, Zalewska T. Sodium butyrate, a Histone Deacetylase inhibitor, exhibits Neuroprotective/Neurogenic effects in a rat model of neonatal hypoxia-ischemia. Mol Neurobiol. 2017;54(7):5300-18. 10.1007/s12035-016-0049-2 PMC5533826 27578020

[8] Siebzehnrubl FA, Buslei R, Eyupoglu IY, Seufert S, Hahnen E, Blumcke I (2007). Histone deacetylase inhibitors increase neuronal differentiation in adult forebrain precursor cells. Exp Brain Res.

[9] Alvarez AA, Field M, Bushnev S, Longo MS, Sugaya K (2015). The effects of histone deacetylase inhibitors on glioblastoma-derived stem cells. J Mol Neurosci.

[10] Fung H, Demple B (2005). A vital role for Ape1/Ref1 protein in repairing spontaneous DNA damage in human cells. Mol Cell.

[11] Domenis R, Bergamin N, Gianfranceschi G, Vascotto C, Romanello M, Rigo S (2014). The redox function of APE1 is involved in the differentiation process of stem cells toward a neuronal cell fate. PLoS One.

[12] Poletto M, Vascotto C, Scognamiglio PL, Lirussi L, Marasco D (2013). Role of the unstructured N-terminal domain of the hAPE1 (human apurinic/apyrimidinic endonuclease-1) in the modulation of its interaction with nucleic acids and NPM1 (nucleophosmin). Biochem J.

[13] Jason M, Kaski JC. Vasodilator therapy: nitrates and Nicorandil. Cardiovasc Drugs Ther. 2016; 10.1007/s10557-016-6668-z. 10.1007/s10557-016-6668-z PMC5658472 27311574

[14] Georgiadis MM, Chen Q, Meng J, Guo C, Wireman R, Reed A (2016). Small molecule activation of apurinic/apyrimidinic endonuclease 1 reduces DNA damage induced by cisplatin in cultured sensory neurons. DNA Repair (Amst).

[15] Zhou Y, Wang Q, Evers BM, Chung DH (2005). Signal transduction pathways involved in oxidative stress-induced intestinal epithelial cell apoptosis. Pediatric Res.

[16] So EC, Chen YC, Wang SC, Wu CC, Huang MC, Lai MS (2016). Midazolam regulated caspase pathway, endoplasmic reticulum stress, autophagy, and cell cycle to induce apoptosis in MA-10 mouse Leydig tumor cells. Onco Targets Ther.

[17] Boland K, Flanagan L, Prehn JH (2013). Paracrine control of tissue regeneration and cell proliferation by Caspase-3. Cell Death Dis.

[18] Amariglio N, Hirshberg A, Scheithauer BW (2009). Donor-derived brain tumor following neural stem cell transplantation in an ataxia telangiectasia patient. PLoS Med.

[19] Radtke C, Redeker J, Jokuszies A (2010). In vivo transformation of neural stem cells following transplantation in the injured nervous system. J Reconstr Microsurg.

[20] Wu W, He Q, Li X, Zhang X, Lu A, Ge R (2011). Long-term cultured human neural stem cells undergo spontaneous transformation to tumor-initiating cells. Int J Biol Sci.

[21] Kaus A, Widera D, Kassmer S, Peter J, Zaenker K, Kaltschmidt C (2010). Neural stem cells adopt tumorigenic properties by constitutively activated NF-kappaB and subsequent VEGF up-regulation. Stem Cells Dev.

[22] Zhao LN, Wang P, Liu YH, Cai H, Ma J, Liu LB, Xi Z, Li ZQ, Liu XB, Xue YX. Mir-383 inhibits proliferation, migration and angiogenesis of glioma-exposed endothelial cells in vitro via vegf-mediated fak and src signaling pathways. Cellular signalling. 2017;30:142-53. 10.1016/j.cellsig.2016.09.007 27693218

[23] Lange C, Garcia MT, Decimo I, Bifari F, Eelen G, Quaegebeur A (2016). Relief of hypoxia by angiogenesis promotes neural stem cell differentiation by targeting glycolysis. EMBO J.

[24] Lim S, Kaldis P (2012). Loss of Cdk2 and Cdk4 induces a switch from proliferation to differentiation in neural stem cells. Stem Cells.

[25] Dokmanovic M, Clarke C, Marks PA (2007). Histone Deacetylase inhibitors: overview and perspectives. Mol Cancer Res.

[26] Lagger G (2002). O’Carro ll D, Rembold M et al. essential function of histone deacetylase 1 in proliferation control and CDK inhibitor repression. EMBO J.

[27] Marks PA, Jiang X (2005). Histone deacetylase inhibitors in programmed cell death and cancer therapy. Cell Cycle.

[28] Marks PA, Xu WS (2009). Histone deacetylase inhibitors: potential in cancer therapy. J Cell Biochem.

[29] Elmi M, Matsumoto Y, Zeng ZJ, Lakshminarasimhan P, Yang W, Uemura A (2010). TLX activates MASH1 forinduction of neuronal lineage commitment of adult hippocampal neuroprogenitors. Mol Cell Neurosci.

[30] Roth SY, Denu JM, Allis CD (2001). Histone acetyltransferases. Annu Rev Biochem.

[31] Gregory PD, Wagner K, Horz W (2001). Histone acetylation and chromatin remodeling. Exp Cell Res.

[32] Thiagalingam S, Cheng KH, Lee HJ, Mineva N, Thiagalingam A, Ponte JF (2003). Histone deacetylases: unique players in shaping the epigenetic histone code. Ann N Y Acad Sci.

[33] Richon VM, Sandhoff TW, Rifkind RA, Marks PA (2000). Histone deacetylase inhibitor selectively induces p21WAF1 expression and gene-associated histone acetylation. Proc Natl Acad Sci U S A.

[34] Hsieh J, Nakashima K, Kuwabara T, Mejia E, Gage FH (2004). Histone deacetylase inhibition-mediated neuronal differentiation of multipotent adult neural progenitor cells. Proc Natl Acad Sci U S A.

[35] Chu W, Yuan J, Huang L, Xiang X, Zhu H, Chen F (2015). Valproic acid arrests proliferation but promotes neuronal differentiation of adult spinal NSPCs from SCI rats. Neurochem Res.

[36] Biermann J, Boyle J, Pielen A, Lagrè WA (2011). Histone deacetylase inhibitors sodium butyrate and valproic acid delay spontaneous cell death in purified rat retinal ganglion cells. Mol Vis.

[37] Sun J, Wang F, Li H, Zhang H, Jin J, Chen W (2015). Neuroprotective effect of sodium butyrate against cerebral ischemia/reperfusion injury in mice. Biomed Res Int.

[38] Valvassori SS, Dal-Pont GC, Steckert AV, Varela RB, Lopes-Borges J, Mariot E (2016). Sodium butyrate has an antimanic effect and protects the brain against oxidative stress in an animal model of mania induced by ouabain. Psychiatry Res.

[39] Georgiadis MM, Luo M, Gaur RK, Delaplane S, Li X, Kelley MR (2008). Evolution of the redox function in mammalian apurinic/apyrimidinic endonuclease. Mutat Res.

[40] Luo M, Zhang J, He H, Su D, Chen Q, Gross ML (2012). Characterization of the redox activity and disulfide bond formation in apurinic/apyrimidinic endonuclease. Biochemist.

[41] Park MS, Kim CS, Joo HK, Lee YR, Kang G, Kim SJ (2013). Cytoplasmic localization and redox cysteine residue of APE1/Ref-1 are associated with its anti-inflammatory activity in cultured endothelial cells. Mol Cells.

[42] Idris NM1, Ashraf M, Ahmed RP, Shujia J, Haider KH (2012). Activation of IL-11/STAT3 pathway in preconditioned human skeletal myoblasts blocks apoptotic cascade under oxidant stress. Regen Med.

[43] Niagara MI, Haider HK, Jiang S, Ashraf M (2007). Pharmacologically preconditioned skeletal myoblasts are resistant to oxidative stress and promote angiomyogenesis via release of paracrine factors in the infarcted heart. Circ Res.

[44] Xiang J, Wan C, Guo R, Guo D. Is hydrogen peroxide a suitable apoptosis inducer for all cell types? Biomed Res Int. Volume 2016, Article ID 7343965, 6-pages. http://dx.doi.org/10.1155/2016/7343965. 10.1155/2016/7343965 PMC4993923 27595106

