# Supplementary material for: Nicorandil potentiates sodium butyrate induced preconditioning of neurons and enhances their survival upon subsequent treatment with H2O2
Source: Transl Neurodegener. 2017 Oct 30;6:29. doi: 10.1186/s40035-017-0097-1 (PMC5662071; doi:10.1186/s40035-017-0097-1)
Supplement: Additional file 1: — (a-b) A conceptual look at how NaB and Nicroandil preconditioning increases neural cell viability upon exposure to oxidative stress. (DOCX 18 kb) [file 40035_2017_97_MOESM1_ESM.docx]

**Nicorandil potentiates sodium butyrate induced preconditioning of neurons and enhances their survival upon subsequent treatment with H_2_O_2_**

**Supplementary data**

**Supplementary Materials and Methods**

**Neural stem cells isolation and culture**

### NSCs were obtained from 14-day old Sprague Dawley rat ganglion eminence as described earlier (Horie N et al 2008). Briefly, the head of embryo was separated and the brain was dissected, the ganglion eminence tissue was pinched out. The collected tissue was transferred to a 15-ml falcon tube contacting DMEM/F12 (Cat #10565018, Invitrogen, Carlsbad, USA), 2% B27 (Cat #17504044, Thermo Fisher Scientific, Waltham, USA), 1% N2 (Cat #17502048, Invitrogen, , Carlsbad, USA), 10ng/ml bFGF (Cat #F029, Sigma, St. Louis, USA), 20 ng/ml EGF (Cat# E9644, St. Louis, USA) and 1% Pen/strep. The tissue was suspended in complete culture media for 2-3 minutes. The suspension was transferred to T-25 c$\boldsymbol{m}^{\boldsymbol{2}}$ and incubated in 37 ̊ centigrade and 5% CO2 for 1 week. One week later some sphere like cell gatherings were formed which called neurospheres. For passaging the neurospheres, the spheres dissociated to single cells using trypsin 0.05%, the single cells were transferred to new culture flasks with concentration 50000 cells/ml.

**Tri-lineage differentiation of neural stem cells**

Single cell suspension of passage# 4 neural stem cells was prepared by treatment with 0.05% trypsin (Cat #25300054, Thermo Fisher Scientific, Waltham, USA). The cells were later cultured on poly-ornithin coated plates (Cat #P3655, Sigma, St. Louis, USA) for 2days. For induction of tri-lineage neural differentiation, 0.5% fetal bovine serum (Cat #26140079, Gibco, Thermo Fisher Scientific, Waltham, USA) was added to the neural stem cells culture medium while concomitantly removing both bFGF and EGF. Three days later, the neural stem cells were differentiated into neurons, oligodendrocytes and astrocytes. To confirm the differentiation of the neural stem cells, immunocytochemistry was performed for MAP-2 (1:1000, cat # ab5392, Abcam, Cambridge, UK), glial fibrillary acidic protein (Cat# Z033401, Dako, Santa Clara, USA) and Oligodendrocyte marker CNPase (cat# ab6319, Abcam, Cambridge, USA) as described earlier (Hosseini SM et al 2015).

### Supplementary References

**1.** Horie N, So K, Moriya T, Kitagawa N, Tsutsumi K, Nagata I, Shinohara K. Effects of oxygen concentration on the proliferation and differentiation of mouse neural stem cells in vitro. Cellular and molecular neurobiology. 2008 Sep 1;28(6):833-45.

2. Hosseini SM, Farahmandnia M, Razi Z, Delavari S, Shakibajahromi B, Sarvestani FS, Kazemi S, Semsar M. Combination cell therapy with mesenchymal stem cells and neural stem cells for brain stroke in rats. International journal of stem cells. 2015 May 31;8(1):99-105.
